# Deciphering the endometrial immune landscape of RIF during the window of implantation from cellular senescence by integrated bioinformatics analysis and machine learning

**DOI:** 10.3389/fimmu.2022.952708

**Published:** 2022-09-05

**Authors:** Xiaoxuan Zhao, Yang Zhao, Yuepeng Jiang, Qin Zhang

**Affiliations:** ^1^ Department of Traditional Chinese Medicine (TCM) Gynecology, Hangzhou Hospital of Traditional Chinese Medicine Affiliated to Zhejiang Chinese Medical University, Hangzhou, China; ^2^ College of Basic Medicine, Hebei College of Traditional Chinese Medicine, Shijiazhuang, China; ^3^ College of Pharmacy, Zhejiang Chinese Medical University, Hangzhou, China

**Keywords:** recurrent implantation failure, immune landscape, cellular senescence, bioinformatics, machine learning

## Abstract

Recurrent implantation failure (RIF) is an extremely thorny issue in *in-vitro* fertilization (IVF)-embryo transfer (ET). However, its intricate etiology and pathological mechanisms are still unclear. Nowadays, there has been extensive interest in cellular senescence in RIF, and its involvement in endometrial immune characteristics during the window of implantation (WOI) has captured scholars’ growing concerns. Therefore, this study aims to probe into the pathological mechanism of RIF from cellular senescence and investigate the correlation between cellular senescence and endometrial immune characteristics during WOI based on bioinformatics combined with machine learning strategy, so as to elucidate the underlying pathological mechanisms of RIF and to explore novel treatment strategies for RIF. Firstly, the gene sets of GSE26787 and GSE111974 from the Gene Expression Omnibus (GEO) database were included for the weighted gene correlation network analysis (WGCNA), from which we concluded that the genes of the core module were closely related to cell fate decision and immune regulation. Subsequently, we identified 25 cellular senescence-associated differentially expressed genes (DEGs) in RIF by intersecting DEGs with cellular senescence-associated genes from the Cell Senescence (CellAge) database. Moreover, functional enrichment analysis was conducted to further reveal the specific molecular mechanisms by which these molecules regulate cellular senescence and immune pathways. Then, eight signature genes were determined by the machine learning method of support vector machine-recursive feature elimination (SVM-RFE), random forest (RF), and artificial neural network (ANN), comprising *LATS1*, *EHF*, *DUSP16*, *ADCK5*, *PATZ1*, *DEK*, *MAP2K1*, and *ETS2*, which were also validated in the testing gene set (GSE106602). Furthermore, distinct immune microenvironment abnormalities in the RIF endometrium during WOI were comprehensively explored and validated in GSE106602, including infiltrating immunocytes, immune function, and the expression profiling of human leukocyte antigen (HLA) genes and immune checkpoint genes. Moreover, the correlation between the eight signature genes with the endometrial immune landscape of RIF was also evaluated. After that, two distinct subtypes with significantly distinct immune infiltration characteristics were identified by consensus clustering analysis based on the eight signature genes. Finally, a “KEGG pathway–RIF signature genes–immune landscape” association network was constructed to intuitively uncover their connection. In conclusion, this study demonstrated that cellular senescence might play a pushing role in the pathological mechanism of RIF, which might be closely related to its impact on the immune microenvironment during the WOI phase. The exploration of the molecular mechanism of cellular senescence in RIF is expected to bring new breakthroughs for disease diagnosis and treatment strategies.

## Introduction

Recurrent implantation failure (RIF) is usually defined as implantation failure in at least three consecutive attempts of *in-vitro* fertilization (IVF), in which one to three high-quality embryos are transferred in each cycle (1, 2). Despite tremendous advances in reproductive medicine, it remains an ongoing conundrum. About 15% of patients seeking IVF treatment are reported to be afflicted by RIF, which is a huge distress and frustration for both patients and clinicians (3). Statistics have shown that approximately 40% of euploid blastocysts fail to implant in transfers, which suggests the desynchronization between the embryo and the endometrium in the window of implantation (WOI) as a potential cause of RIF (4, 5). During the WOI period, the endometrium becomes poised to transition to a pregnant state, featured as differentiation of endometrial stromal cells (EnSCs) into decidual cells (DCs) and change of the influx and function of immunocytes in the local endometrium (6). Perturbations of these key cellular and molecular biological events tend to induce the breakdown of the feto–maternal interface and RIF (7, 8), but the underlying mechanisms are poorly understood. Accumulated evidence has suggested a conspicuous link between cellular senescence in peri-implantation endometrium and RIF (9, 10). Cellular senescence is a state of permanent cell cycle arrest and manifests with a prominent secretion of various bioactive molecules, including reactive oxygen species, pro-inflammatory cytokines, chemokines, and growth factors, called senescence-associated secretory phenotype (SASP) (11, 12). SASP secreted by senescent cells creates a long-lasting and highly disordered pro-inflammatory response, which also attracts multiple immunocytes, thus jointly facilitating an unfavorable microenvironment for embryo implantation (13, 14). Single-cell transcriptomics revealed that decidualized assembloids harbored senescent subpopulations, and senescence in the stroma calibrated the emergence of anti-inflammatory decidual cells and pro-inflammatory senescent decidual cells, which controlled endometrial fate decisions at implantation and was closely correlated with RIF (15). Moreover, intricate immune regulation is one of the most crucial aspects for the successful implantation of the hemiallogenic embryo (16, 17). Conversely, inappropriate immunocytes as well as immune function are implicated in RIF as evidenced by increasing high-quality studies (18). Furthermore, to date, compelling evidence has also indicated that senescent cells are capable of regulating the immune microenvironment and contributing to advances in pathological mechanisms in various diseases (19–21). However, the understanding of cellular senescence, immune infiltration landscape, and the linkages between the two in the context of RIF disease is still close to blank.

Nowadays, the revolutionary development of microarray technology and bioinformatics greatly facilitates the development of biomedicine. A tremendous amount of high-throughput data is piling up in public databases, which greatly helps with uncovering the potential etiopathogenesis and identifying candidate targets for drug design (22). Machine learning has recently been widely applied to learn the representation of high-dimensional features derived from gene expression data on account of its powerful capabilities in classification (23, 24). The ingenious combination of bioinformatics analysis and machine learning is a creative and crucial way to establish novel diagnostic models and understand pathological mechanisms at the molecular level, which is in line with the latest research trends. However, the application of this method in RIF is still blank, and much of the potential valuable information remains to be uncovered.

**Figure 1:**
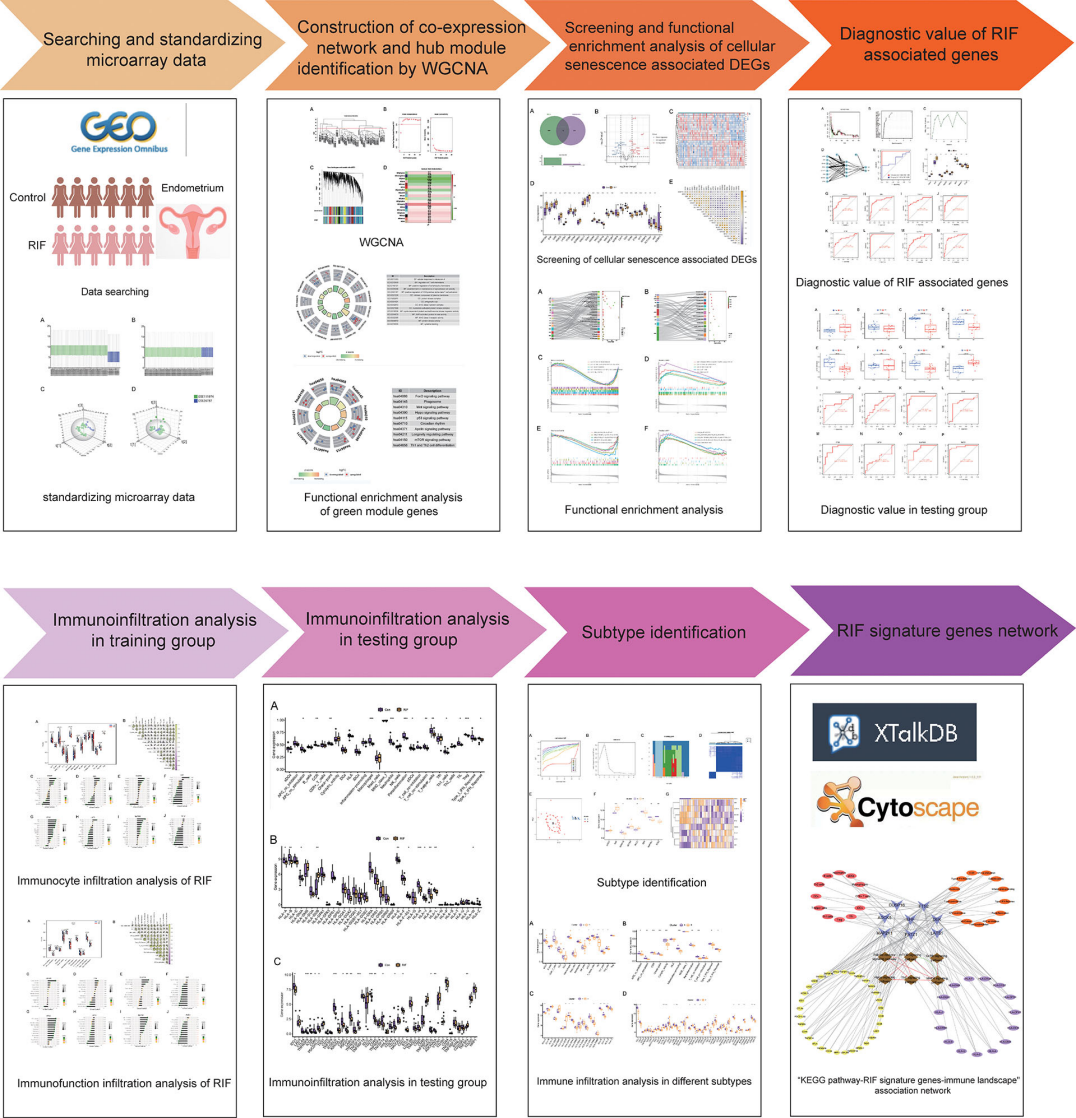
The flow diagram of the study.

In this study, RIF raw microarray data were acquired from the Gene Expression Omnibus (GEO) database, and cellular senescence-related genes were obtained from the Cell Senescence (CellAge) database for systematic analysis. Firstly, the enrichment analysis of key module genes obtained by the weighted gene co-expression network analysis (WGCNA) was carried out. Subsequently, differentially expressed genes (DEGs) related to cellular senescence were screened. Gene Ontology (GO) and the Kyoto Encyclopedia of Genes and Genomes (KEGG) pathway and gene-set enrichment analysis (GSEA) were conducted to further explore the biological mechanisms that the cellular senescence-associated DEGs were involved in. Moreover, we screened and validated the signature genes by machine learning algorithms of support vector machine-recursive feature elimination (SVM-RFE), random forest (RF), and artificial neural network (ANN). After that, we analyzed the difference in immune landscape between the RIF group and the normal group by assessing infiltrating immunocytes, immune function, and the expression profiling of HLA (human leukocyte antigen) genes and immune checkpoint genes. Moreover, we evaluated the correlation between the signature genes and immune landscape in RIF. Last but not least, we clustered RIF samples into two distinct subtypes according to the signature genes and compared the endometrial immunity between subtypes. The workflow chart is shown in **Figure 1**. To the best of our knowledge, this study is the first to explore the pathological mechanisms of RIF in terms of cellular senescence affecting the endometrial immune landscape during the WOI stage by integrated bioinformatics analysis and machine learning, which provides new insights into the treatment strategy for RIF and offers valuable insulation and foundation for further innovative studies on RIF.

## Materials and methods

### Data collection

The microarray datasets were systematically extracted from the GEO database (https://www.ncbi.nlm.nih.gov/geo/) (25) with the keyword “Recurrent implantation failure.” Datasets that met the following inclusion criteria were included: 1) *Homo sapiens*; 2) expression profiling by array; 3) the experiment included patients with RIF and fertile controls; 4) the sample size was at least 10 people, with at least 5 patients in each group; and 5) the sample was from the endometrium during WOI. Finally, three datasets were included (GSE26787, GSE111974, and GSE106602), of which GSE26787 and GSE111974 were taken as the training set, and GSE106602 was taken as the testing set. The details of the three datasets are shown in **Table 1**. Furthermore, 279 cellular senescence-associated genes were downloaded from the CellAge database (https://genomics.senescence.info/cells/) (26).

**Table 1:** Basic information of the included dataset.

GSE no.	No. of samples	Platform	Description	Country	Type
GSE26787	5 vs. 5	Affymetrix Human Genome U133 Plus 2. 0 Array	Endometrial biopsy was performed in the non-conceptional cycle in the middle luteal phase of RIF and healthy fertile women (controls).	France	Training set
GSE111974	24 vs. 24	Agilent-039494 SurePrint G3 Human GE v2 8 × 60K Microarray 039381	24 patients with RIF treated at the IVF clinic and 24 fertile control patients recruited from the gynecology clinic of Istanbul University School of Medicine during 2014–2015 were involved in this prospective cohort study.	Turkey	Training set
GSE106602	16 vs. 19	Illumina HiSeq 2500	We compared the mid-secretory transcriptome profiles from healthy women with the profiles of women with repeated IVF failure to find transcriptome changes related to problems with endometrial receptivity.	Estonia	Testing set

### Data preprocessing and normalization

The raw data were downloaded from the GEO database, and then preprocessed and normalized by R statistical software (version 4.1.2, https://www.r-project.org/) and Bioconductor analysis tools (http://www.bioconductor.org/). The “affy” R language package was applied to conduct RMA background correction, complete log2 transformation, quantile normalization, and median polish algorithm summarization. Probes without matching gene symbols were excluded. For multiple probes mapped to the same gene, the mean value was taken as the final expression value. The results are shown in box plots and three-dimensional PCA cluster diagrams before and after normalization (**Supplementary Figures 1A–D**).

### Construction of the co-expression network and hub module identification by WGCNA

The WGCNA is a widely used method to uncover critical interacted genetic modules and key genes by linking gene networks to clinical traits. In this study, the WGCNA co-expression system was established by using the “WGCNA” package in R software (27) with DEGs from the GSE26787 and GSE111974 datasets, with normal control and RIF as clinical features. First, genes with variation higher than 25% across samples in the combined dataset were selected as the input dataset for the subsequent WGCNA. Then, the outlier cases were removed by hierarchical clustering analysis with the “goodSamplesGenes” function. After that, the appropriate soft threshold was determined by using the pickSoftThreshold function and validated by the correlation between *k* and *p*(*k*). Subsequently, the correlation matrix was converted into an adjacency matrix, which was further processed into a topological overlap matrix (TOM). The dynamic tree cutting approach was performed to identify various modules. The relationship between these modules and RIF was investigated. Finally, the module with the greatest Pearson correlation coefficient was picked for further investigation.

### Identification of cellular senescence-associated DEGs

We used the processed data to filter DEGs by using the “limma” packages of R software (version 4.1.2) with the screening criteria of adjusted *P*-value <0.05 and |log2 fold change (FC)| >0.5 (28). The Venn online mapping tool was used to screen for intersections of DEG and cellular senescence-associated genes, namely, cellular senescence-associated DEGs, which were visualized with heatmaps and volcano plots by the “ggplot2” package in R software (29).

### Function enrichment analysis of cellular senescence-associated DEGs

The cellular senescence-associated DEGs were imported into the WebGestalt website (http://www.webgestalt.org/) (30) for GO and KEGG pathway enrichment analysis. The species was selected as “*Homo sapiens*,” and the reference set was selected as genome protein-coding. Items with a *P*-value <0.05 were displayed in a bubble plot by the “ggplot2” package in R software. Moreover, GSEA (version 4.1.0) on cellular senescence-associated DEGs was conducted by using the “c2.cp.kegg.v7.0.symbols.gmt” and “c5.go.v7.4.symbols” gene set derived from the Molecular Signatures Database (MSigDB; version 7.1) as reference (31). The threshold for significant terms was adjusted *q* value <0.05.

### Screening cellular senescence-associated signature genes of RIF by machine learning

The SVM-RFE algorithm and RF were utilized to screen the cellular senescence-associated signature genes in RIF. SVM-RFE is a sequence backward selection algorithm based on the maximum margin principle of SVM, which has superior classification performance for high-dimensional datasets (32). It was first proposed in gene selection (33) and was applied to gene selection in cancer classification. After that, the SVM-RFE algorithm was further improved to improve its performance and efficiency, and it has been widely used in gene expression data analysis, protein function prediction, image detection, and other fields (34). In this study, the SVM-RFE algorithm was implemented by using the package of “e1071,” “kernlab,” and “caret” in R software for feature dimensionality reduction. The RF algorithm is an ensemble method that combines many decision trees and makes a single decision on behalf of the ensemble by combing the results of multiple classifiers together (35). Each decision tree in the forest is built by using the bootstrap technique to select various samples from the original dataset and then training it with a feature set chosen by the random bagging mechanism (36). Decisions made by a large number of distinct individual trees are then voted on, and the class with the most votes as a result of the voting is assigned as the class prediction (37). Here, we used the RF algorithm to predict RIF with the input of cellular senescence-associated signature genes by the “randomForest” package in R software. In addition, we constructed an ANN model for the feature genes obtained from the above method according to the gene score by using the packages of “neuralnet” and “neuralnettools” in R software. The artificial neural network can simulate the structure and function of the brain neural network and deduce a set of classification rules from a set of disordered and irregular data, so as to realize the correct classification and construct a high-accuracy diagnosis model (38). Furthermore, the receiver operating characteristic (ROC) curve was utilized to evaluate the accuracy of the ANN model in the training and testing sets.

Finally, feature genes screened by the above machine learning methods were regarded as signature genes of RIF from the aspect of cellular senescence, namely, RIF signature genes. We observed the expression of these eight genes in the training set (GSE26787 and GSE111974) and the testing set (GSE106602). Furthermore, the ROC curve was constructed by the “pROC” package in R software to evaluate the prediction accuracy of these feature genes.

### The tissue localization and function of RIF signature genes

The information of tissue localization of RIF signature genes was obtained through the BioGPS (building your own mash-up of gene annotations and expression profiles) website (http://biogps.org) (39) and the Human Protein Atlas (https://www.proteinatlas.org/) (40). Moreover, to further perceive the functions of these genes, we searched the databases of BioGPS, GeneCards (https://www.genecards.org/) (41), Alliance of Genome Resources (https://www.alliancegenome.org/) (42), and UniProt (https://www.uniprot.org/) (43).

### Immune infiltration landscape analysis of RIF and its correlation with cellular senescence-associated signature genes

Single-sample gene-set enrichment analysis (ssGSEA) is an extension of the GSEA method, which allows to analyze the pathways enriched by genes in each sample, thereby analyzing the activation degree of specific pathways (44, 45). Here, ssGSEA was performed by using the “GSVA” package in R software to explore the immune cell-related pathways in RIF patients and healthy controls. Moreover, the expression of HLA molecules and immune checkpoints was quantified and compared between groups. Furthermore, correlation analysis between cellular senescence-associated signature genes and immunocytes, immune functions, HLA, and immune checkpoints in RIF was evaluated by Spearman correlation analysis with the “corrplot” package in R software. *P <*0.05 was considered statistically significant.

### Consensus clustering analysis

Consensus clustering analysis was carried out by using the “ConsensusClusterPlus” package of R software on the basis of the expression profiling of cellular senescence-associated signature genes (46). The above step was implemented for 1,000 iterations for guaranteeing the robustness of classification. The Euclidean distance between specimens was determined. Consensus cluster analysis was conducted with a maximum number of clusters of 9, 50 repeats, a sample proportion of 0.8, and a ratio of features to samples of 1. The pam cluster algorithm was selected, and cluster analysis was conducted by using Euclidean as a distance function. After the completion of cluster analysis, PCA analysis was conducted by using the “limma” package, and classification dot plots were drawn to verify the cluster results. In addition, the expression of RIF-specific cell senescence-related genes, immunocytes, immune functions, HLA, and immune checkpoints in the different subtypes were analyzed.

### Construction of the “KEGG pathway–RIF signature genes–immune landscape” association network

In order to intuitively understand the molecular and biological processes involved and communicated by RIF signature genes, we constructed the “KEGG pathway–RIF signature genes–immune landscape” association network. First, the cross-talk relationship between the KEGG pathways enriched by RIF signature genes was obtained through the XTalkDB website (http://www.xtalkdb.org/contactus) (47). In addition, we sorted out the correspondence between the RIF signature genes and the significantly correlated immunocytes, immune function, HLA moleculars, and immune checkpoints. Next, we used Cytoscape 3.7.1 software to visualize the “KEGG pathway–RIF signature genes–immune landscape” association network.

## Results

### Establishment of a co-expression network and hub module identification

Module detection was performed by hierarchical clustering and dynamic tree cut functions (**Supplementary Figures 2A–C**). The soft thresholding power was set at 4 according to the scale independence and mean connectivity values. A total of 17 modules were divided by WGCNA and were identified by different colors. Among these modules, the green module showed the most significant correlation with RIF (*r* = −0.67, *P* = 0.003) (**Supplementary Figure 2D**). Therefore, we focused on the biological functions of the genes in the green module through GO and KEGG analysis. The results of GO annotation suggested that these genes were mostly enriched in processes involved in cellular response to interleukin-4, regulation of T-cell chemotaxis, positive regulation of lymphocyte chemotaxis, etc. As for the KEGG pathway, the majority of genes were enriched in the Wnt signaling pathway, p53 signaling pathway, and longevity regulating pathway, which were closely related to the regulation of cell fate decision. Based on the above results, it intimately implied that the pathological mechanism of RIF was bound up with immune abnormalities and the dysregulation of cell fate (**Supplementary Figures 2E, F**).

### Identification and integrative analysis of DEGs

**Figure 2:**
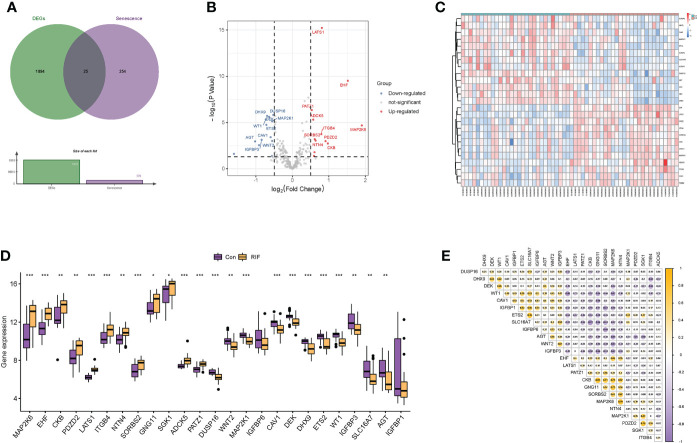
Identification of differentially expressed genes related to cellular senescence. **(A)** Venn diagram of differentially expressed genes and genes related to cellular senescence. **(B, C)** Volcano plot and heatmap visualized the differentially expressed genes (DEGs) related to cellular senescence. In the volcano plot, each dot represents a gene. The red plot points represent upregulated genes, and the blue plot points represent downregulated genes, and in the heatmap, each row represents a DEG related to cellular senescence, and each column represents a sample. **(D)** The difference in the mRNA expression profiling of cellular senescence-associated DEGs between the recurrent implantation failure (RIF) and control groups. (**E**) The correlations between cellular senescence-associated DEGs in the RIF group. * represents *P <*0.05 compared with the control group, ** represents *P <*0.01 compared with the control group, and *** represents *P <*0.001 compared with the control group.

After preprocessing and normalization of the included datasets, differential expression analysis was performed. A total of 1,919 DEGs were obtained with the screening conditions of *P*-value <0.05 and |log2 FC| >0.5. Then, 25 overlapped genes were obtained by the Venn diagram after intersecting the 1,919 DEGs with 279 cellular senescence-associated genes derived from the CellAge database (**Figure 2A**). Moreover, these 25 genes were taken as a new gene set for further study, namely, cellular senescence-associated DEGs. Next, we observed the difference in the mRNA expression profiling of these 25 genes between the RIF and control groups. Among the 25 cellular senescence-associated DEGs, a total of 12 genes were upregulated and 13 genes were downregulated (**Figures 2B–D**). Subsequently, we explored the correlations between cellular senescence-associated DEGs through the Spearman correlation test. The results corroborated that *CKB* was significantly positively correlated with *GNG11* (*r* = 0.81, *P* < 0.05), and *SLC16A7* was significantly negatively correlated with *CKB* (*r* = −0.77, *P* < 0.05) (**Figure 2E**).

### Functional enrichment analysis

To comprehend the biological processes these cellular senescence-associated DEGs were involved in, we performed the GO and KEGG enrichment analysis and GSEA method. As shown in the GO enrichment analysis, those genes were mainly enriched in the biological process of cell growth, regulation of growth, negative regulation of canonical Wnt signaling pathway, etc. As for the cellular component, these genes were mainly enriched in insulin-like growth factor binding protein complex, growth factor complex, and cell–substrate junction. Furthermore, as for the molecular function, these genes were mainly enriched in insulin-like growth factor binding, protein serine/threonine/tyrosine kinase activity, MAP kinase activity, etc. (**Supplementary Figure 3A**).

The results of the KEGG enrichment analysis signified that the 25 cellular senescence-associated DEGs were significantly activated in aging-related pathways, such as mTOR signaling pathway, cellular senescence, etc., and immune inflammation-related pathways, such as Toll-like receptor signaling pathway, TNF signaling pathway, etc. The intricate mapping relationship between genes and pathways was also displayed in the Sankey diagram in which *MAP2K1* corresponded to the most KEGG pathways, indicating a crucial role in communicating the complicated cross-talk between different pathways (**Supplementary Figure 3B**). Furthermore, we applied the GSEA method to appraise the differences in activation of GO enrichment and KEGG pathway between the RIF and fertile control samples. Our results revealed that GO terms of immune response, leukocyte migration, and T-cell activation were significantly activated in fertile control samples, while cytoplasmic pattern recognition receptor signaling pathway was activated in RIF samples (**Supplementary Figures 3C, D**). Meanwhile, the KEGG pathways of chemokine signaling pathway, cytokine–cytokine receptor interaction, and natural killer cell-mediated cytotoxicity pathway exhibited increased activation in fertile control samples, while abc transporters and steroid biosynthesis pathways were activated in RIF samples (**Supplementary Figures 3E, F**).

### Screening cellular senescence-associated signature genes of RIF by machine learning

**Figure 3:**
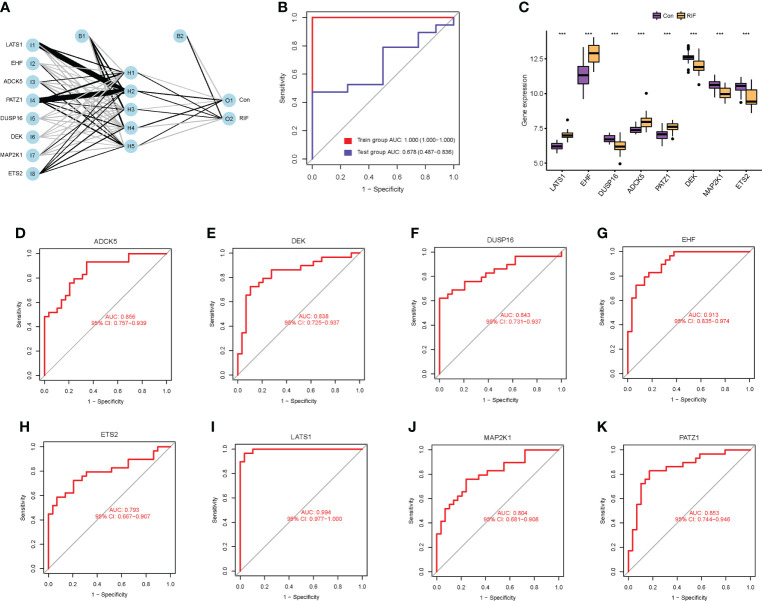
Diagnostic value of cellular senescence-associated DEGs in RIF. **(A)** The neural network model: I1–I8 are the input layers (the score and weight of eight RIF signature genes), H1–H5 are the hidden layers, and O1–O2 are the output layers (sample attributes). **(B)** The receiver operating characteristic (ROC) curves for evaluating the diagnostic efficacy of the neural network model in the GSE26787 and GSE111974 (training set) and GSE106602 (testing set). **(C)** The mRNA expression profiling analysis of the eight RIF signature genes. **(D–K)** The ROC curves of the RIF signature genes. *** represents *P <*0.001 compared with the control group.

**Figure 4:**
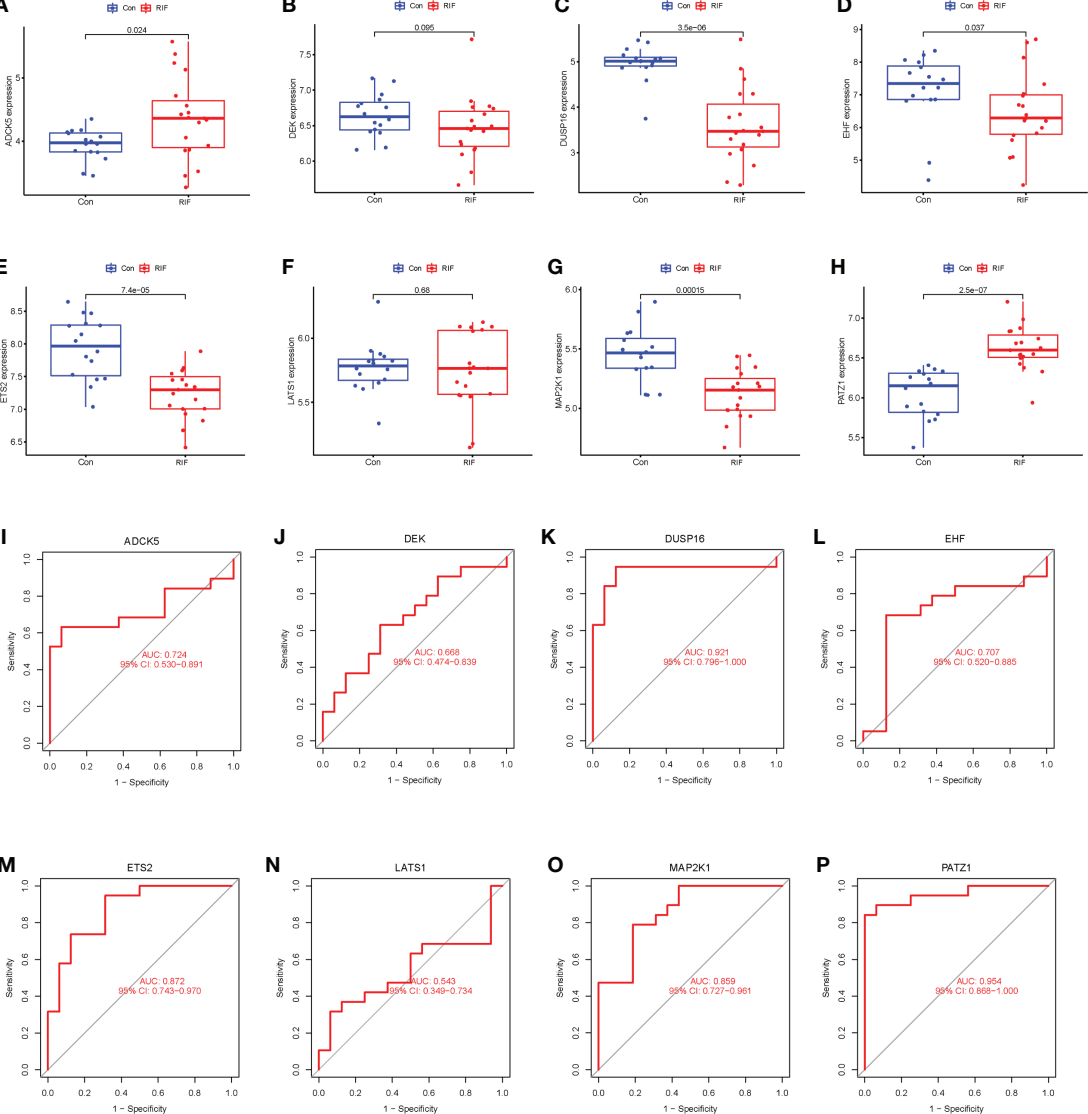
External validation to further test the diagnostic performance of the RIF signature genes. **(A–H)** Expression differences of the RIF signature genes among different groups in the testing set. **(I–P)** The ROC curves of the RIF signature genes in the testing set.

To clarify the diagnostic value of cellular senescence-associated DEGs, we performed machine learning of SVM-RFE algorithms and RF algorithms and obtained two and eight feature genes, respectively (**Supplementary Figures 4A–C**). After combining the results of the two algorithms, a total of eight cellular senescence-associated signature genes of RIF were obtained, namely, *LATS1*, *EHF*, *DUSP16*, *ADCK5*, *PATZ1*, *DEK*, *MAP2K1*, and *ETS2*. Moreover, these eight genes were used for constructing the neural network, and the results showed that the eight genes could distinguish the control samples from the RIF samples well, with an accuracy rate of 100%. Furthermore, ROC was constructed, and the results showed that the AUC of the training set and the testing set was 1.000 and 0.678, respectively, which demonstrated the high accuracy of the ANN model (**Figures 3A, B**). Thus, the eight genes were named as RIF signature genes. Among these genes, *LATS1*, *EHF*, *ADCK5*, and *PATZ1* were highly expressed in RIF samples (*P* < 0.001), and *DUSP16*, *DEK*, *MAP2K1*, and *ETS2* declined significantly in RIF samples (*P* < 0.001) (**Figure 3C**). To further evaluate the diagnosis value of the eight feature genes screened by the above machine learning methods, we constructed the ROC curves of these eight RIF signature genes and calculated the AUC. The results elucidated that the AUC ranged from 0.793 to 0.994 (**Figures 3D–K**), indicating a good diagnostic performance of these genes. In addition, we performed external validation with the testing dataset of GSE106602. The mRNA expression profiling of the eight RIF signature genes was evaluated, and the result indicated that the mRNA levels of *ADCK5*, *DUSP16*, *EHF*, *ETS2*, *MAP2K1*, and *PATZ1* were significantly different between the RIF and fertile control groups (*ADCK5*, *EHF*: *P* < 0.05; *DUSP16*, *ETS2*, *MAP2K1*, *PATZ1*: *P* < 0.001) (**Figures 4A–H**). Then, we constructed the ROC curves and calculated the AUC of these eight genes. The AUC varied from 0.543 to 0.954 (**Figures 4I–P**), which also confirmed the excellent diagnostic performance of these genes.

### The tissue localization and function of RIF signature genes

To further perceive the tissue localization and function of these genes, we searched the databases of BioGPS, Human Protein Atlas, GeneCards, Alliance of Genome Resources, and UniProt to acquire comprehensive information, as shown in **Table 2**. From this table, we could discover that multiple genes were expressed in the uterine tissue and immunocytes. This was the premise that they could participate in cellular senescence, regulate immunization activities, and thus, affect endometrial receptivity.

**Table 2:** Immune localization of RIF signature genes.

Gene	Description (referring to the GeneCards database)	Expression in uterine tissue (referring to the Human Protein Atlas database)	Expression in immunocytes (referring to the Human Protein Atlas and BioGPS databases)	Subcellular summary (referring to the Human Protein Atlas database)	Function (referring to the BioGPS, GeneCards, Alliance of Genome Resources, and UniProt database)
*LATS1*	Large tumor suppressor kinase 1	Yes	NK cell	–	*LATS1* is a negative regulator of YAP1 in the Hippo signaling pathway, inhibiting its phosphorylation and translocation into the nucleus, thereby regulating cell proliferation, cell death, and cell migration (PMID: 22898666). It is also involved in controlling the expression of p53 (PMID: 28644436).
*EHF*	ETS homologous factor	Yes	Macrophage, DC, CD4^+^ T cell, CD8^+^ T cell	Nucleoplasm, Golgi apparatus	*EHF* acts as a transcriptional repressor, modulates the nuclear response to mitogen-activated protein kinase signaling cascades, and may be involved in epithelial differentiation and carcinogenesis (PMID: 27612480).
*DUSP16*	Dual specificity phosphatase 16	Yes	CD4^+^ T cell, Treg cell, neutrophil	Nucleoplasm	*DUSP16* is a dual specificity protein phosphatase involved in the inactivation of MAP kinases. It can dephosphorylate MAPK10 bound to ARRB2 and regulate the c-Jun amino-terminal kinase (JNK) and extracellular signal-regulated kinase (ERK) pathways (PMID: 11489891).
*ADCK5*	aarF domain containing kinase 5	Yes	Monocyte, DC, CD4^+^ T cell	Plasma membrane, cytosol	*ADCK5* is predicted to enable protein serine/threonine kinase activity, is involved in protein phosphorylation, and is an integral component of the membrane (provided by the Alliance of Genome Resources, April 2022).
*PATZ1*	POZ/BTB and AT hook containing zinc finger 1	Yes	Monocyte, DC, CD4^+^ T cell, CD8^+^ T cell, neutrophil, B cell	Nucleoplasm	Transcriptional regulator that plays a role in many biological processes such as embryogenesis, senescence, T-cell development, or neurogenesis (PMID: 10713105, PMID: 25755280, PMID: 31875552). It interacts with the TP53 protein to control genes that are important in the proliferation and in the DNA damage response. Mechanistically, the interaction inhibits the DNA binding and transcriptional activity of TP53/p53 (PMID: 25755280).
*DEK*	DEK proto-oncogene	Yes	Gamma delta T cell, Treg cell	Nucleoplasm, cytosol	*DEK* is involved in chromatin organization (PMID: 17524367).
*MAP2K1*	Mitogen-activated protein kinase kinase 1	Yes	Monocyte, DC	Plasma membrane, cytosol	As an essential component of MAP kinase signal transduction pathway, this kinase is involved in many cellular processes such as proliferation, differentiation, transcription regulation, and development (PMID: 8388392, PMID: 9465908).
*ETS2*	ETS proto-oncogene 2, transcription factor	Yes	Monocyte	Nucleoplasm, plasma membrane, cytosol	*ETS2* can bind specifically the DNA GGAA/T core motif in gene promoters and stimulate transcription (PMID: 11909962).

### Immune infiltration landscape of RIF and its correlation with RIF signature genes

**Figure 5:**
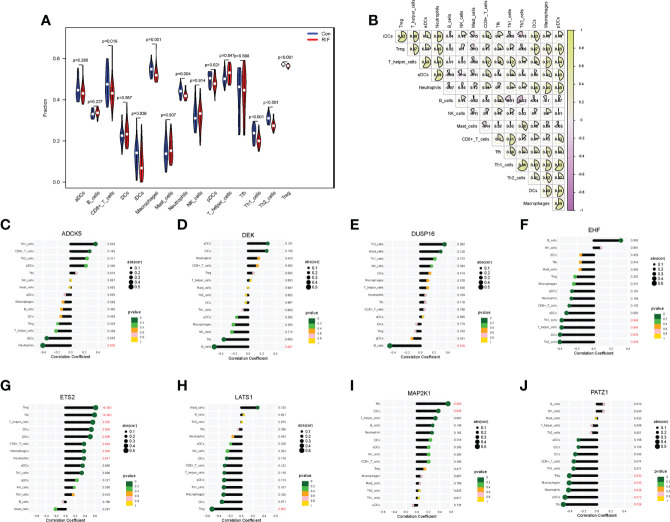
Immune cell infiltration landscape of RIF and its correlation with the RIF signature genes. **(A)** The distribution of immunocytes between the RIF and control samples. **(B)** The correlation heatmap showed the correlation between different immunocytes in RIF samples. (**C–J**) The lollipop chart showed the correlation between RIF signature genes and immunocytes in RIF samples.

To fully grasp the immune landscape of RIF, we analyzed differences in immune cells, immune function, and expression profiling of the HLA gene set and immune checkpoints between patients with RIF and fertile controls. Regarding the types of immunocytes, the levels of CD8^+^ T cells, immature dendritic cells (iDCs), macrophages, neutrophils, plasmacytoid dendritic cells (pDCs), Th1 cells, Th2 cells, and Treg in the RIF group were significantly lower than those in the control group (*P* < 0.05). Meanwhile, the T helper cells were significantly increased in the RIF group (*P* = 0.047) (**Figure 5A**). Furthermore, the changes of macrophages, pDCs, neutrophils, Th1 cells, and Treg cells in RIF in the testing set were consistent with the training set (**Supplementary Figure 5A**). The above data indicated the heterogeneity in immunocytes between control and RIF samples. In addition, Spearman correlation analysis implied that Th1 cells were significantly positively correlated with Th2 cells in RIF samples (*r* = 0.66, *P* < 0.05), and B cells were significantly negatively correlated with Th2 cells (*r* = −0.32, *P* < 0.05) (**Figure 5B**). Moreover, we further investigated the interactions of RIF signature genes with infiltrated immunocyte in RIF samples. We obtained the most correlated pairs of the RIF signature gene–immunocyte, including *ADCK5* and neutrophils (*r* = −0.40, *P* < 0.05), *DEK* and B cells (*r* = −0.49, *P* < 0.01), *DUSP16* and B cells (*r* = −0.45, *P* < 0.05), *EHF* and Th2_cells (*r* = −0.40, *P* < 0.05), *ETS2* and follicular helper T cells (Tfh) (*r* = 0.59), Treg (*r* = 0.62, *P* < 0.001), *LATS1* and Treg (*r* = −0.51, *P* < 0.01), *MAP2K1* and Tfh (*r* = 0.54, *P* < 0.01), and *PATZ1* and Tfh (*r* = −0.49, *P* < 0.01) (**Figures 5C–J**).

**Figure 6:**
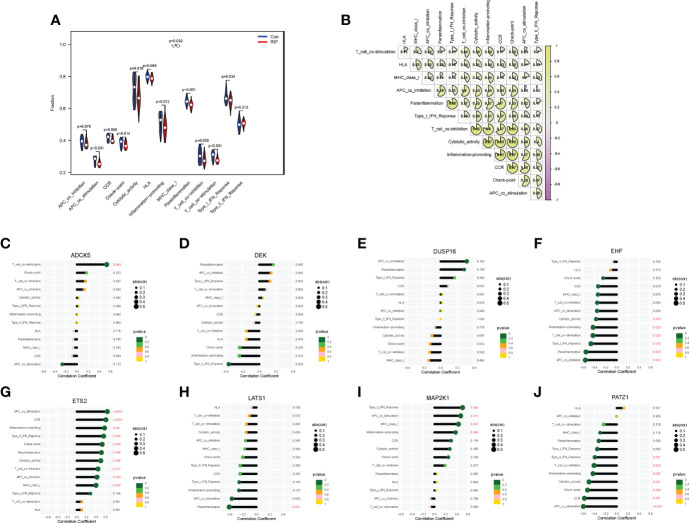
Immune function landscape of RIF and its correlation with RIF signature genes. **(A)** The distribution of immune functions between RIF and control samples. **(B)** Correlation heatmap showed the correlation between different immune functions in RIF samples. **(C–J)** The lollipop chart showed the correlation between RIF signature genes and immune functions in RIF samples.

When it came to the comparison of immune functions between groups, the results substantiated that the functions of APC co-stimulation, CCR, checkpoint, cytolytic activity, inflammation-promoting, MHC class I, parainflammation, T-cell co-inhibition, T-cell co-stimulation, and type I IFN response in the RIF group were significantly lower than those in the control group (*P* < 0.05) (**Figure 6A**). Moreover, the changes of APC co-stimulation, CCR, checkpoint, parainflammation, and T-cell co-stimulation in RIF in the testing set were consistent with the training set (**Supplementary Figure 5A**). The above data corroborated the heterogeneity in immune function between the control and RIF samples. In addition, Spearman correlation analysis revealed that the function of checkpoint was positively correlated with CCR (*r* = 0.87, *P* < 0.05) and inflammation-promoting (*r* = 0.87, *P* < 0.05) in RIF samples (**Figure 6B**). Furthermore, we obtained the immune functions that were most associated with the RIF signature genes by Spearman correlation analysis. The most strongly associated pairs included *ADCK5* and T_cell_co-stimulation (*r* = 0.53, *P* < 0.01), *EHF* and parainflammation (*r* = −0.55, *P* < 0.01), *ETS2* and CCR (*r* = 0.60, *P* < 0.001), APC co-stimulation (*r* = 0.63, *P* < 0.001), *LATS1* and parainflammation (*r* = −0.40, *P* < 0.05), *MAP2K1* and type II IFN response (*r* = 0.50, *P* < 0.01), and *PATZ1* and APC co-stimulation (*r* = −0.59, *P* < 0.001) (**Figures 6C–J**).

**Figure 7:**
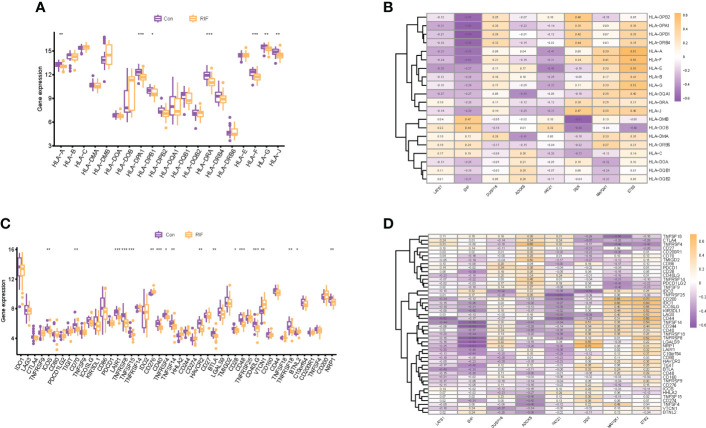
Human leukocyte antigen (HLA) and immune checkpoint of RIF and its correlation with RIF signature genes. **(A, C)** The distribution of HLA and immune checkpoint between RIF and control samples. **(B, D)** The correlation heatmap showed the correlation between RIF signature genes, HLA genes, and immune checkpoints in RIF samples. * represents *P* < 0.05 compared with the control group,**represents *P* < 0.01 compared with the control group, ***represents *P* < 0.001 compared with the control group.

When it came to the HLA gene set expression profile, the expression levels of *HLA-A*, *HLA-G*, *HLA-J* (*P* < 0.01), *HLA-DPB1* (*P* < 0.05), *HLA-DPA1*, *HLA-DRA*, and *HLA-F* (*P* < 0.001) in the RIF group were all significantly decreased (**Figure 7A**). Moreover, the expression profile changes of *HLA-A* and *HLA-J* in RIF in the testing set were consistent with the training set (**Supplementary Figure 5B**). The results of the Spearman correlation analysis illuminated that *ETS2* was significantly positively correlated with *HLA-A* (*r* = 0.63, *P* < 0.05), and *EHF* was significantly negatively correlated with *HLA-DPB1* (*r* = −0.64, *P* < 0.05) in the RIF group (**Figure 7B**).

As for the immune checkpoints, the mRNA expression profiles of *ICOS* (*P* < 0.01), *CD70* (*P* < 0.01), *LAIR1* (*P* < 0.001), *TNFRSF8* (*P* < 0.001), *TNFSF15* (*P* < 0.001), *CD40* (*P* < 0.001), *TNFRSF4* (*P* < 0.05), *TNFSF14* (*P* < 0.01), *HAVCR2* (*P* < 0.01), *BTLA* (*P* < 0.01), *CD28* (*P* < 0.05), *CD48* (*P* < 0.001), *CD40LG* (*P* < 0.001), *TNFRSF18* (*P* < 0.01), and *NRP1* (*P* < 0.01) in the RIF group were significantly lower than those in the control group (*P* < 0.05), and the mRNA expression of *CD276* (*P* < 0.01), *VTCN1* (*P* < 0.01), and *BTNL2* (*P* < 0.05) was significantly increased in the RIF group when compared with the control group (*P* < 0.05) (**Figure 7C**). Furthermore, the expression profile changes of *ICOS*, *LAIR1*, *TNFSF15*, *CD40*, *TNFSF14*, and *CD40LG* in RIF in the testing set were consistent with the training set (**Supplementary Figure 5C**). The results of the Spearman correlation analysis displayed that *DEK* was significantly positively correlated with *NRP1*, *ETS2*, and *CD200* (*r* = 0.70, *P* < 0.05) in the RIF group, and *EHF* was significantly negatively correlated with *TNFRSF8* and *CD244* (*r* = −0.64, *P* < 0.05) in the RIF group (**Figure 7D**).

### Immune infiltration landscape of distinct subtypes

**Figure 8:**
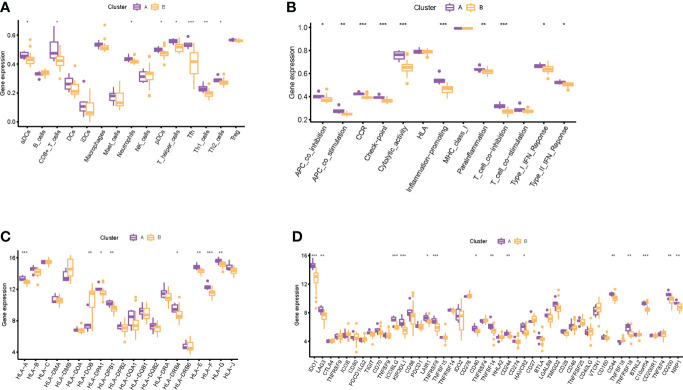
Immune-related infiltration landscape in RIF by unsupervised clustering based on the eight RIF signature genes. **(A)** The abundance differences of different immunocytes between the two subtypes. **(B)** The activity differences of different immune functions between the two subtypes. **(C)** The abundance differences of different HLA between the two subtypes. **(D)** The abundance differences of different immune checkpoints between the two subtypes. * represents *P <*0.05 compared with cluster A, ** represents *P <*0.01 compared with cluster A, and *** represents *P <*0.001 compared with cluster A.

Through consensus clustering analysis, we clustered RIF samples into two subtypes based on the mRNA expression profiling of the eight RIF signature genes, namely, subtype A (*n* = 6) and subtype B (*n* = 23) (**Supplementary Figures 6A–E**). Except for *LATS1*, *ADCK5*, and *DEK*, the expression differences of the remaining five RIF signature genes among the groups were statistically significant (*P* < 0.05) (**Supplementary Figures 6F, G**). In order to figure out the differences of immune infiltration landscape between the two subtypes, the ssGSEA method was utilized to quantify the infiltrating level of immunocytes and immune function, and the expression profiling of the HLA and immune checkpoint gene sets was also evaluated. The results showed that various infiltrating immunocytes in cluster B were significantly reduced when compared with cluster A, such as anchorage-dependent cells (aDCs), CD8^+^ T cells, neutrophils, pDCs, T helper cells, etc. (*P* < 0.05) (**Figure 8A**). The immune functions in cluster B, such as APC co-inhibition, APC co-stimulation, CCR, checkpoint, etc., were significantly decreased (*P* < 0.05) (**Figure 8B**). In addition, the mRNA expression profiles of *HLA-A*, *HLA-DPA1*, *HLA-DPB1*, etc. in cluster B were significantly decreased, and HLA-DOB was significantly increased when compared with cluster A (*P* < 0.05) (**Figure 8C**). Furthermore, various immune checkpoints in cluster B were also significantly decreased, such as *TNFRSF9*, *CD70*, *LAIR*, etc. (**Figure 8D**). All the above results indicated that different expression patterns of RIF signature genes exerted a distinct impact on the immune infiltration landscape in the endometrium during WOI.

## Discussion

Embryo implantation remains the rate-limiting step for IVF, and endometrial receptivity during the WOI has received extensive attention, which has been considered as a crucial impetus for deciphering the pathological mechanism of RIF (48, 49). Currently, in addition to the recognized immune factors, accumulated evidence has also highlighted the significant influence of cellular senescence on endometrial receptivity (50, 51). For instance, Deryabin et al. uncovered that premature senescence of EnSC could alter the “meta-signature” of human endometrial receptivity and affect embryo invasion based on an *in-vitro* implantation model (52). Moreover, studies by Chen et al. corroborated that premature aging of the endometrium existed in young women with RIF and was closely related to implantation failures (53). Furthermore, cellular senescence has been demonstrated to be closely related to the immune microenvironment (54). Senescence subjects the immune system to constant immune stressors and inflammatory assaults, which in turn promotes immune senescence and is intimately tied to diverse pathological changes (55). Now, the association of cellular senescence and immune regulation has attracted growing attention in various diseases. Relevant findings suggest that SASP factors contribute to disease progression by regulating immunity and doing harm to the tissue homeostasis (56, 57). However, the effect of cellular senescence on the immune system of RIF has not been elucidated. Studies have confirmed that multiple SASP factors promote the activation of molecular signaling cascades that are associated with embryo rejection (58–60). These discoveries provide us with a sufficient basis to demonstrate the potential linkage between cellular senescence and immune regulation in RIF.

Nowadays, the current diagnosis of the disease is retrospectively established based on repeated failed attempts in IVF-ET, which pose great challenges to clinical strategy formulation for precise prevention and treatment of RIF. In this study, we interpreted the pathological mechanism of RIF from a new perspective and explored a new strategy for the diagnosis of RIF based on bioinformatics analysis combining machine learning. Overall, we probed the involvement of cellular senescence in the pathological mechanism of RIF, revealed the immune infiltration landscape in the endometrium during WOI, and analyzed the correlation between cellular senescence and immune dysregulation. After a series of explorations, we obtained the following significant findings: 1) through the WGCNA method, the genes in the most relevant modules of RIF mainly affected immune functions (T-cell chemotaxis, positive regulation of lymphocyte chemotaxis) and cell fate decision (Wnt signaling pathway, p53 signaling pathway, longevity regulating pathway). 2) Through differential expression analysis, we obtained 25 cellular senescence-associated DEGs of RIF and verified their specific molecular mechanisms in regulating cellular senescence and immunity through functional enrichment analysis. 3) A total of eight RIF signature genes (*LATS1*, *EHF*, *DUSP16*, *ADCK5*, *PATZ1*, *DEK*, *MAP2K1*, and *ETS2*) were screened by machine learning of SVM-RFE, RF, and ANN. In addition, we accessed the BioGPS, Human Protein Atlas, GeneCards, Alliance of Genome Resources, and UniProt databases to analyze the localization and function of these eight RIF signature genes and discovered that they were all expressed in uterine tissue and immunocytes. Through findings 1–3, we preliminarily concluded that the pathological mechanism of RIF was related to cellular senescence and abnormal immune regulation. Moreover, senescence-associated DEGs exhibited the potential as RIF biomarkers, which also suggested the close relationship between cellular senescence and RIF. 4) We then analyzed the endometrial immune landscape during WOI in patients with RIF and fertile controls, including infiltrating immunocytes, immune function, HLA gene sets, and immune checkpoints, as well as evaluated their correlation with RIF signature genes. 5) In addition, we also performed consensus clustering analysis according to the RIF signature genes, and the differences in the endometrial immune landscape between subtypes also suggested the correlation between cellular senescence and immune regulation. Through findings 4–5, we could further conclude that cellular senescence was closely associated with the abnormal endometrial immunoregulation during WOI in RIF.

**Figure 9:**
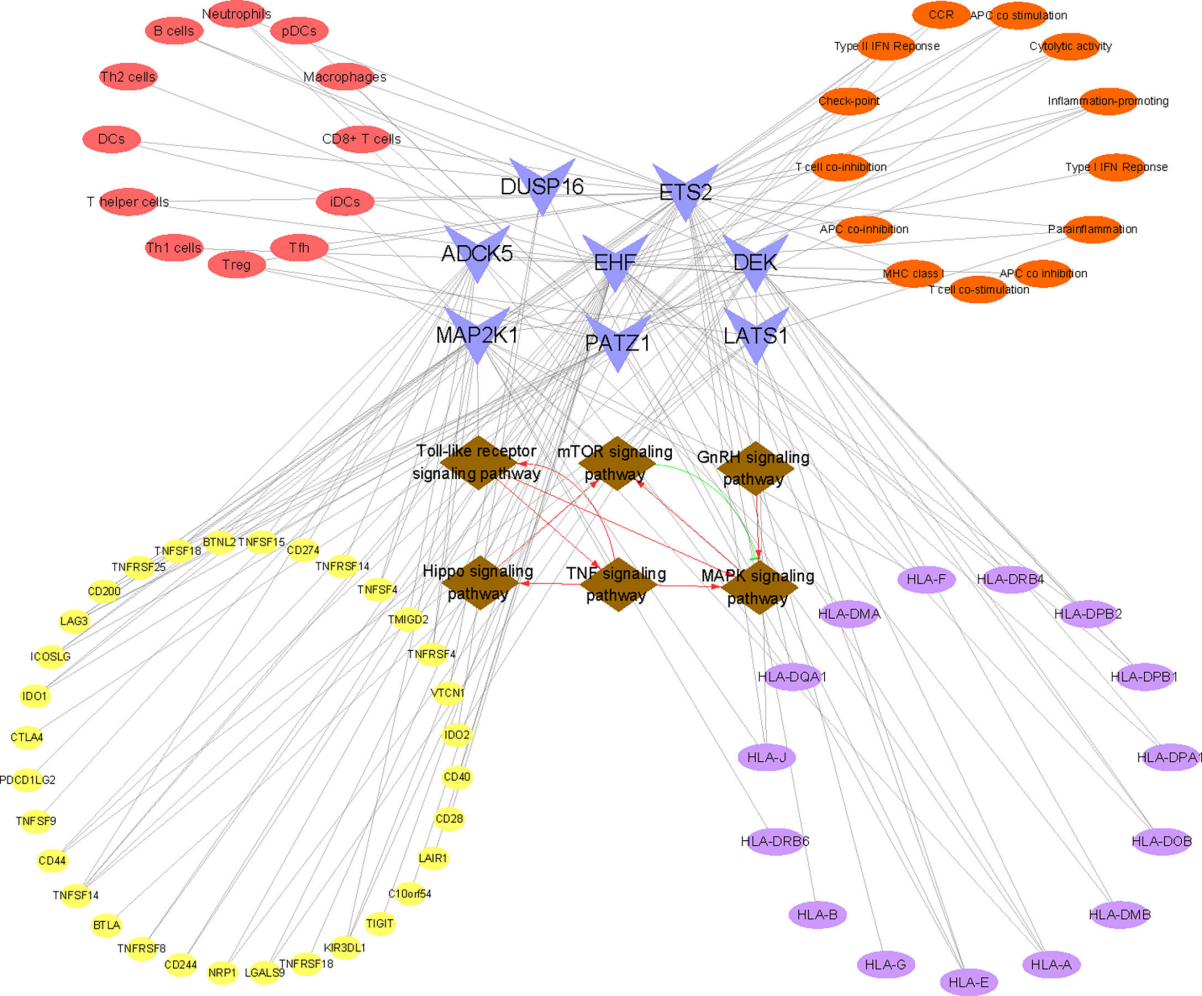
Construction of the “KEGG pathway–RIF signature genes–immune landscape” association network. The network consists of 86 nodes and 160 edges. Brown nodes represent the KEGG pathway, blue nodes represent RIF signature genes, red nodes represent immune cell type, orange nodes represent immune function, purple nodes represent HLA-related genes, and yellow nodes represent immune checkpoint-related genes. The black lines represent the relationships between nodes. The red lines represent facilitation effects between pathways, and the green lines represent inhibition between pathways.

By integrating the above results, the following key indications deserved to be explored in depth. First, we found that *MAP2K1* was one of the significantly downregulated cellular senescence genes in RIF both in the training set and the testing set and was involved in the most signal transduction, such as MAPK signaling pathway, Hippo signaling pathway, etc. Although *MAP2K1* has not attracted enough attention in RIF yet, the detection of *MAP2K1* has been regarded as a vital biomarker for evaluating cellular senescence in various diseases (61, 62). Therefore, this study suggested that we could focus on *MAP2K1*, which might bring new discoveries for the diagnosis and treatment of RIF from the aspect of cellular senescence in the future. In addition, among the various pathways regulated by *MAP2K1*, we take the MAPK signaling pathway as an example. Studies have confirmed that *MAP2K1* encodes a dual specificity protein kinase which lies upstream of MAP kinases and stimulated the enzymatic activity of MAP kinases upon a wide variety of extra- and intracellular signals. Moreover, the MAP kinase/ERK cascade has been verified to be inactive in senescent cells, which is capable of significantly reducing many proinflammatory components of the SASP (63, 64). Fernandes et al. also demonstrated that MAP kinase/ERK rewired in senescent cells rendering them phenotypically different (65). Furthermore, studies implied that MAPK influenced cell survival in the endometrium (66). From the aforementioned analysis, we hypothesized that the decreased mRNA profiling of *MAP2K1* in RIF tended to restrain the activation of the MAPK signaling pathway, thus procuring cellular senescence and impairing endometrial receptivity. In addition, to intuitively comprehend the involvement of the eight signature genes in the biologic process, the mapping relationship between genes and signaling pathways was visualized in **Figure 9**. The six signaling pathways communicated by RIF signature genes interacted with each other and formed a complex biological network, which provided abundant clues for follow-up research.

When it comes to immunity and pregnancy, it is universally acknowledged that immune tolerance to the semi-allogeneic fetus is significantly rudimentary for a successful pregnancy. Abnormalities of endometrial immunity are involved in the pathogenesis of RIF (67, 68). At present, research on the immune microenvironment in the endometrium of RIF during WOI is in full swing. However, due to the complex composition of immunocytes and the complicated cross-talk of immune functions, traditional research methods fail to systematically reflect the immune landscape in the endometrium. This time, we utilized ssGSEA to analyze 15 types of infiltrated immunocytes and 13 types of immune functions in RIF and control groups and also compared the expression of HLA molecules and immune checkpoints between groups, both in the training and validation sets, respectively. The above analysis revealed that the immune landscape of RIF was significantly different from fertile controls. Take the Treg lymphocyte as an example. It is a pivotal class of immunosuppressive cells, expresses forkhead box P3 (Foxp3), and secretes the anti-inflammatory cytokines, such as TGF-β1 and IL-10, which can suppress the excessive immune response induced by the fetus, playing a pushing role in maintaining an immune tolerance microenvironment during pregnancy (69, 70). Moreover, higher levels of endometrial Treg cells have been confirmed to be a positive prognostic factor for improving pregnancy outcome in IVF (71). Our results manifested that Treg cells were reduced in patients with RIF when compared with the control group, which was consistent with multiple studies (72–74). However, the reason for the reduction of Treg cells in RIF was still perplexing.

We speculated that the disorder of immunocytes and immune function was related to cellular senescence. Continuing with the example of reduced Treg cells in RIF, our study illuminated that Treg cells were significantly negatively correlated with *LATS1* and positively correlated with *ETS2*. *LATS1* is the core kinases of Hippo effector Yes-associated protein 1 (YAP1), which can lead to the phosphorylation and inhibition of YAP1 (75), thus regulating cell proliferation, differentiation, and boosting cellular senescence (76–78). As for *ETS2*, it is a member of the ETS family of DNA-binding transcription factors. Studies have suggested that *ETS2* can bind to the proximal promoter of human telomerase reverse transcriptase (hTERT) and positively regulate hTERT, thus inhibiting cellular senescence (79). Through the above analysis, we conjectured that the increase of *LATS1* and the decrease of *ETS2* in RIF may reduce the number of local endometrial Treg cells by promoting cellular senescence. Given that the above gene expression is not tissue specific, we cannot determine in which cells senescence occurs. According to published studies, we assumed that there were three possibilities: 1) the increase of senescent decidual cells leads to an excess of IL-6 and TGF-β, the most common SASP factors, which can induce the differentiation of CD4^+^ T cells to Th17 and reduce the level of Treg cells (80, 81). 2) The clearance mechanism of senescent cells by immunocytes is abnormal. Senescent cells are subject to immune surveillance by multiple components of the immune system (82), especially immunocytes with the function of phagocytosis. Moreover, impaired immune surveillance can lead to senescent cell accumulation (83, 84). Studies have affirmed that immunocytes exert a pivotal role in endometrial fate decisions at implantation (85). One of the principal functions of macrophages is phagocytic clearance of senescent cells (86, 87). However, we observed a decreased level of endometrial macrophages in RIF patients both in training and testing sets, which may account for the accumulation of senescent cells. Moreover, senescent cells attract and activate immune cells and serve as highly immunogenic targets for immune clearance, the microenvironment of which may prompt the Treg/Th17 balance to tilt toward the Th17 cell. 3) The senescence of CD4^+^ T lymphocytes themselves leads to the reduction of Treg cells. As a cellular counterpart, CD4^+^ T lymphocytes can also undergo cellular senescence, which could also contribute to an immune response biased toward Th17 lymphocytes from Treg lymphocytes (88). Now, senescent T cells are corroborated to be accumulated in aging, chronic viral infections, and autoimmune disorders, which are also considered as potential targets for disease treatment (89–91). However, the current research on immunosenescence in RIF disease is still blank. To systematically and intuitively grasp the correlation between immune landscape and cellular senescence in RIF, we visualized the association of immune cells, immune function, HLA molecules, and immune checkpoints with the eight cellular senescence-related signature genes of RIF in **Figure 9**. Taken together, our findings undoubtedly threw innovative light on the mechanism of an abnormal immune microenvironment in RIF, which also contributed to uncovering potential intervention targets for drug design.

## Conclusion

In conclusion, our study is the first to explore the involvement of cellular senescence in the pathological mechanism of RIF at the molecular level based on bioinformatics combined with machine learning strategy. This study signified that cellular senescence was a potential pathological mechanism of RIF, and cellular senescence-associated genes were expected to serve as novel diagnostic biomarkers for RIF. Moreover, cellular senescence was involved in the regulation of the endometrial immune microenvironment in RIF during WOI, which provided a groundbreaking direction for the exploration of the pathogenesis of abnormal immunity in RIF. At present, our understanding of cellular senescence is only at the tip of the iceberg, and knowledge about its correlation with the pathological mechanism of RIF is still quite finite. Combining cellular senescence with the regulation of the immune microenvironment landscape to reveal the pathogenic mechanism of RIF is revolutionary for the research of RIF, which makes up for the blank in the field of immune mechanism in RIF to a great extent. This study will also encourage more researchers to carry out senescence-related research in the field of RIF. However, this study also has some limitations, which we must admit. Firstly, the results of this study are based on bioinformatics analysis. Although verified by external testing datasets, its accuracy needs to be further substantiated by experiments. Secondly, immune infiltration analysis in this study uses the most widely used ssGSEA method to quantify the number of immunocytes, but single-cell sequencing is still necessary to obtain the most accurate information. Last but not least, this study only found the correlation between cellular senescence and abnormal immune microenvironment in the endometrium of RIF during WOI, but the cause and effect between the two calls for further exploration. In conclusion, all our findings confirm the involvement of cellular senescence in RIF and its close correlation with the immune characteristics of RIF, which provide new insights into the pathogenesis, diagnosis, and treatment of RIF.

## Data availability statement

The original contributions presented in the study are included in the article/**Supplementary Material**. Further inquiries can be directed to the corresponding author.

## Author contributions

XZ and QZ: conceptualization. XZ and YZ: data curation. YJ and QZ: data analysis. XZ and YZ: writing—original draft. QZ: reviewed the manuscript. All authors read and approved the final article.

## Funding

This study was supported by the Research on the Secondary Development of Jianpi Antai Mixture (2021C03080).

## Acknowledgments

Special thanks to Yuepeng Jiang from the Zhejiang Chinese Medical University for his contribution to the data analysis.

## Conflict of interest

The authors declare that the research was conducted in the absence of any commercial or financial relationships that could be construed as a potential conflict of interest.

## Publisher’s note

All claims expressed in this article are solely those of the authors and do not necessarily represent those of their affiliated organizations, or those of the publisher, the editors and the reviewers. Any product that may be evaluated in this article, or claim that may be made by its manufacturer, is not guaranteed or endorsed by the publisher.

## Supplementary material

Supplementary Figure 1 Data preprocessing and normalization. The box plots of the GSE26787 and GSE111974 datasets before **(A)** and after **(B)** normalization. Three- dimensional PCA cluster plot of the GSE26787 and GSE111974 datasets before **(C)** and after **(D)** normalization.Green nodes represent GSE111974 and blue nodes represent GSE26787.

Supplementary Figure 2 Construction of WGCNA modules. **(A)** Sample dendrogram of control and RIF group. **(B)** Scale independence and mean connectivity under diverse soft-thresholding powers. **(C)** Co-expression modules for each gene under the hierarchical clustering tree are assigned diverse colors. The dynamic tree cutcorresponds to the original modules, and merged dynamic corresponds to the merged modules finally identified. **(D)**Heatmap of the relationships between co-expression modules and clinical traits. The number indicates the correlation coefficients between co-expression modules and clinical traits, and the number in parentheses indicates the corresponding p-values. **(E)**GO enrichment analysis of genes in the green module. **(F)**KEGG enrichment analysis of genes in the green module.

Supplementary Figure 3 Function enrichment of cellular senescence associated DEGs. **(A, B)** Sankey-bubble diagram showing the GO and KEGG enrichment analysis of cellular senescence associated DEGs. **(C)** GSEA results for the activation of GO enrichment in control samples. **(D)** GSEA results for the activation of GO enrichment in RIF samples. **(E)** GSEA results for the activation of KEGG enrichment in control samples. **(F)** GSEA results for the activation of KEGG enrichment in RIF samples.

Supplementary Figure 4 Machine learning of SVM-RFE and RF algorithms for clarifying the diagnostic value of cellular senescence associated DEGs. **(A)**Random forest of cellular senescence associated DEGs. X-axis represents the number of trees,Y-axis represents the error of cross validation, green curve represents the error of control group, red curve represents the error of RIF group, and black curve represents the error of all samples. **(B)**MeanDecreaseGini coefficient diagram: X-axis is MeanDecreaseGini value, which is used to judge the classification of the model; Y-axis is gene name. In this study, genes corresponding to MeanDecreaseGini≥1 were selected. **(C)**SVM-REFscreening feature genes: X-axis represents the change of gene number, Y-axis represents the error of cross validation.

Supplementary Figure 5 Immune related infiltration landscape in testing set. **(A)**The distribution of immunocytes and immune functions between RIF and control samples in testing set. **(B)**The abundance differences of different HLA between RIF and control samples in testing set. **(C)**The abundance differences of different immune checkpoint between RIF and control samples in testing set. * represents *P*<0.05 compared with the control group,**represents *P*<0.01 compared with the control group, ***represents *P*<0.001 compared with the control group.

Supplementary Figure 6 Identifying 2 distinct subtypes in RIF by unsupervised clustering based on 8 RIF signature genes. **(A)** The consensus clustering cumulative distribution function (CDF) for k=2-9. **(B)** The relative change in area under the CDF curve for k=2-9. **(C)** Item tracking plot showing the consensus cluster of items (in column) at each k (in row). **(D)** Consensus matrix plots depicting consensus values on a white to blue color scale ordered by consensus clustering when k= 2. **(E)** t-SNE plots confirming the classification accuracy of two distinct subtypes across RIF samples. **(F–G)** Box diagram and heatmap showed the expression level of 8 RIF signature genes in the two subtypes. * represents *P*<0.05 compared with the cluster A,**represents *P*<0.01 compared with the cluster A, ***represents *P*<0.001 compared with the cluster A.

## Abbreviations

AUC, area under the ROC; CDF, cumulative distribution function; DEGs, differentially expressed genes; GSEA, gene set enrichment analysis; IVF, *in-vitro* fertilization; RF, random forest; RIF, recurrent implantation failure; ROC, receiver operating characteristic curve; ssGSEA, single-sample gene-set enrichment analysis; SVM-RFE, support vector machine-recursive feature elimination; WGCNA, weighted gene co-expression network analysis.
