# Supplementary figures and images for: Deciphering the endometrial immune landscape of RIF during the window of implantation from cellular senescence by integrated bioinformatics analysis and machine learning
Source: Front Immunol. 2022 Sep 5;13:952708. doi: 10.3389/fimmu.2022.952708 (PMC9484583; doi:10.3389/fimmu.2022.952708)

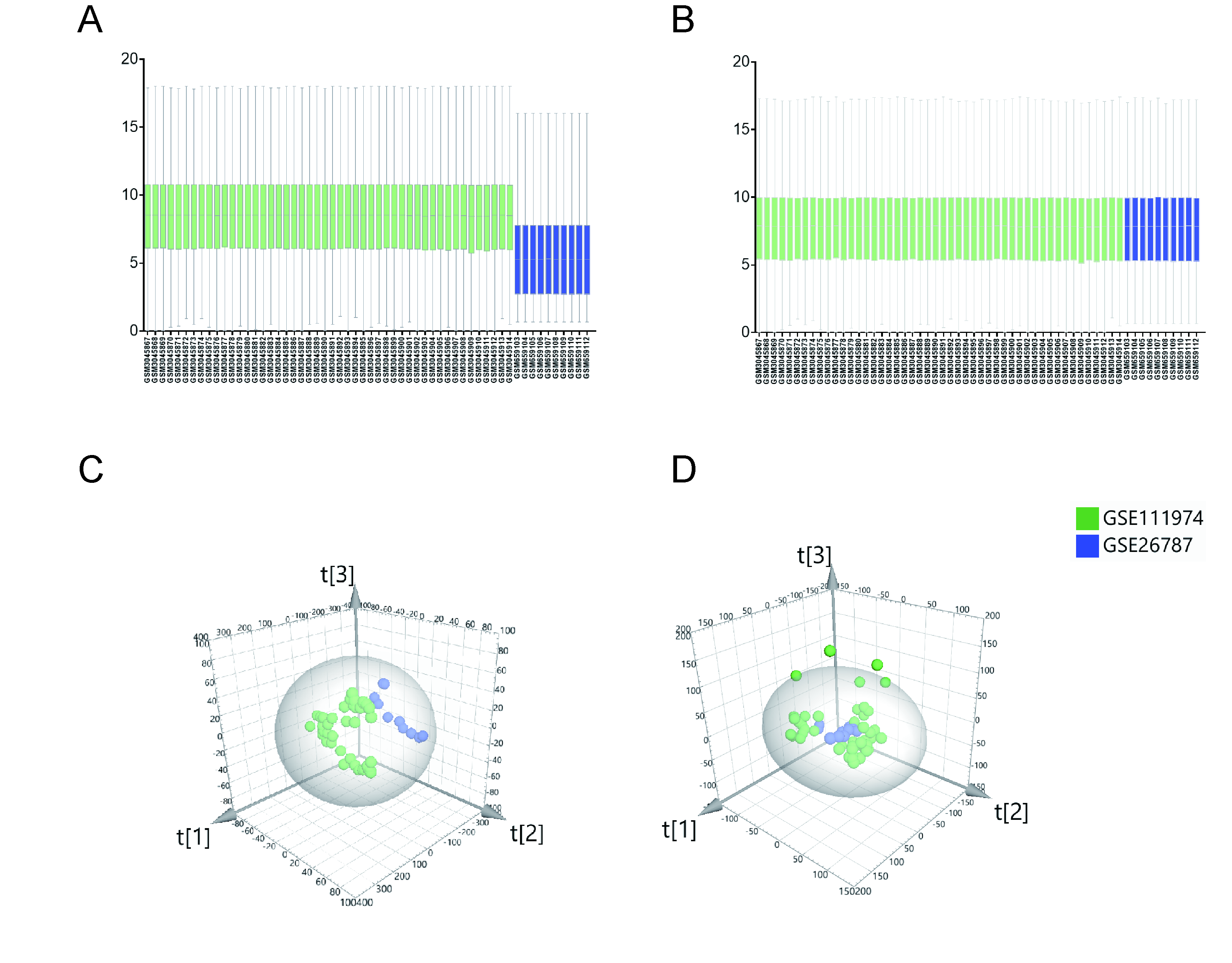

Supplement: Supplementary Figure 1 — Data preprocessing and normalization. The box plots of the GSE26787 and GSE111974 datasets before (A) and after (B) normalization. Three- dimensional PCA cluster plot of the GSE26787 and GSE111974 datasets before (C) and after (D) normalization.Green nodes represent GSE111974 and blue nodes represent GSE26787. [file Image_1.tif]

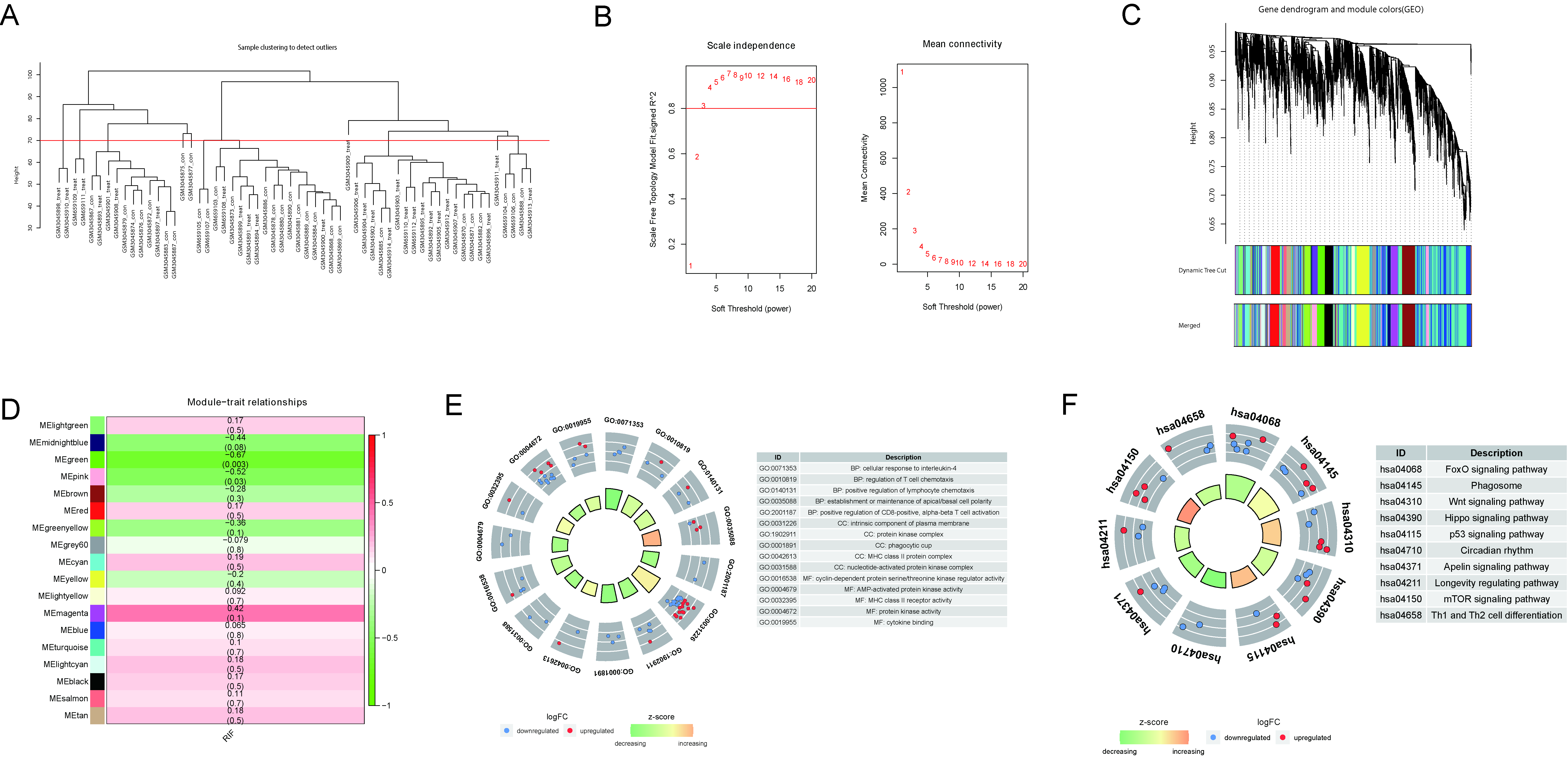

Supplement: Supplementary Figure 2 — Construction of WGCNA modules. (A) Sample dendrogram of control and RIF group. (B) Scale independence and mean connectivity under diverse soft-thresholding powers. (C) Co-expression modules for each gene under the hierarchical clustering tree are assigned diverse colors. The dynamic tree cutcorresponds to the original modules, and merged dynamic corresponds to the merged modules finally identified. (D)Heatmap of the relationships between co-expression modules and clinical traits. The number indicates the correlation coefficients between co-expression modules and clinical traits, and the number in parentheses indicates the corresponding p-values. (E)GO enrichment analysis of genes in the green module. (F)KEGG enrichment analysis of genes in the green module. [file Image_2.tif]

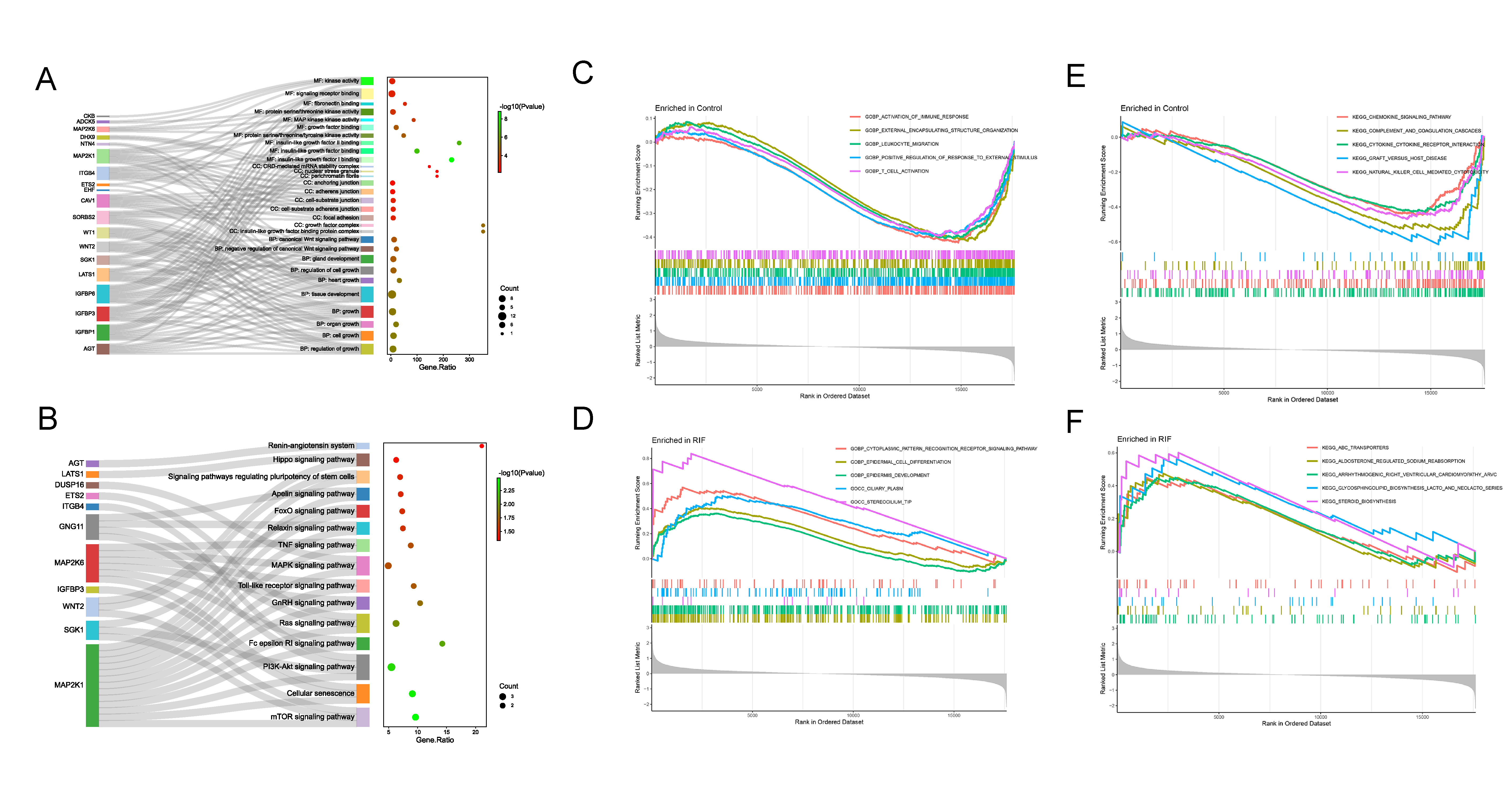

Supplement: Supplementary Figure 3 — Function enrichment of cellular senescence associated DEGs. (A, B) Sankey-bubble diagram showing the GO and KEGG enrichment analysis of cellular senescence associated DEGs. (C) GSEA results for the activation of GO enrichment in control samples. (D) GSEA results for the activation of GO enrichment in RIF samples. (E) GSEA results for the activation of KEGG enrichment in control samples. (F) GSEA results for the activation of KEGG enrichment in RIF samples. [file Image_3.tif]

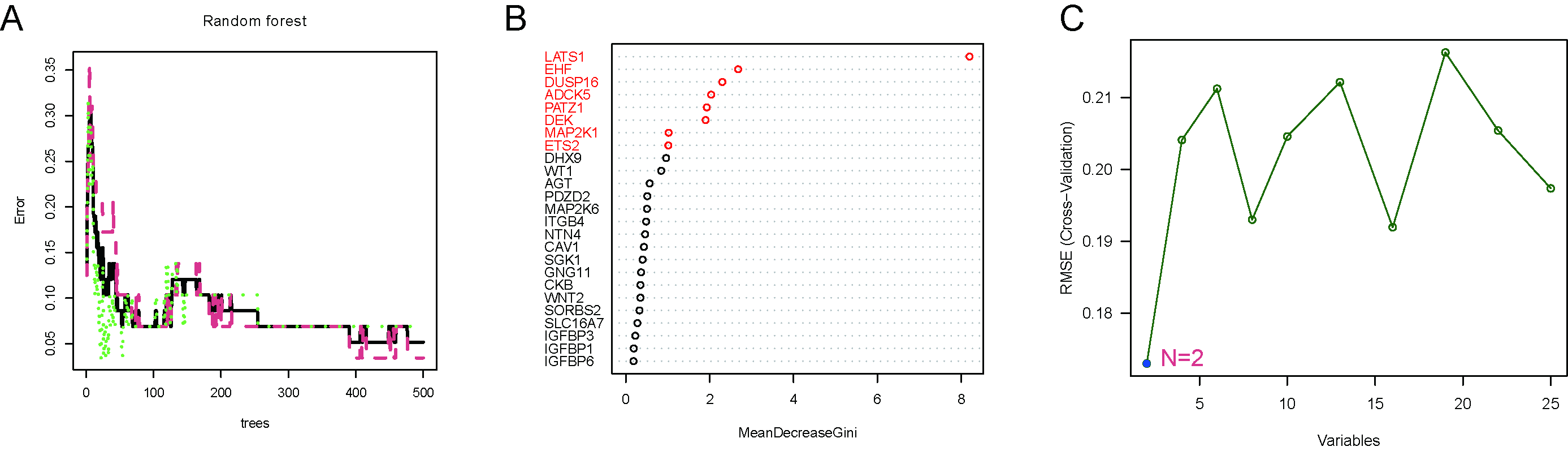

Supplement: Supplementary Figure 4 — Machine learning of SVM-RFE and RF algorithms for clarifying the diagnostic value of cellular senescence associated DEGs. (A)Random forest of cellular senescence associated DEGs. X-axis represents the number of trees,Y-axis represents the error of cross validation, green curve represents the error of control group, red curve represents the error of RIF group, and black curve represents the error of all samples. (B)MeanDecreaseGini coefficient diagram: X-axis is MeanDecreaseGini value, which is used to judge the classification of the model; Y-axis is gene name. In this study, genes corresponding to MeanDecreaseGini≥1 were selected. (C)SVM-REFscreening feature genes: X-axis represents the change of gene number, Y-axis represents the error of cross validation. [file Image_4.tif]

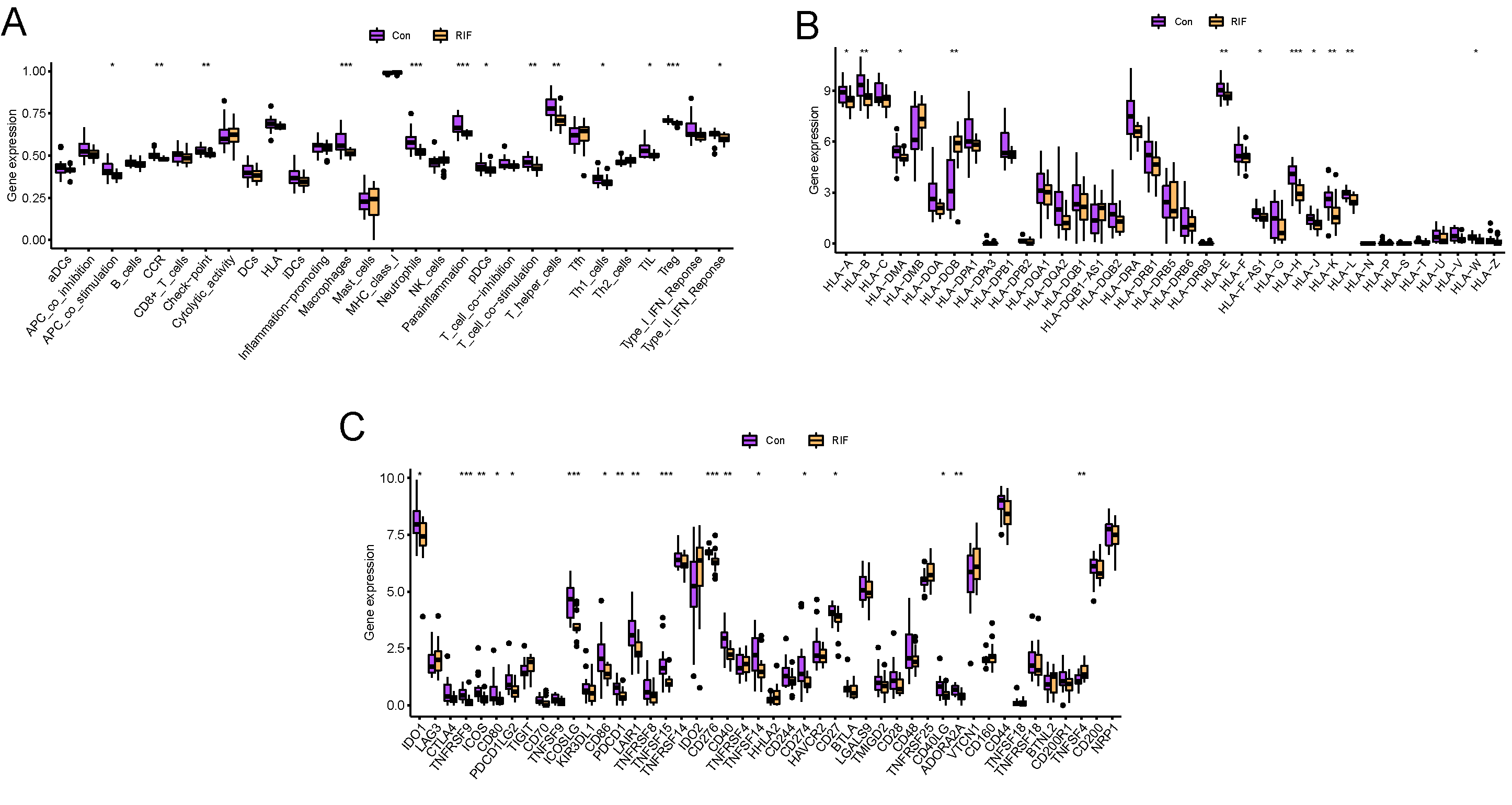

Supplement: Supplementary Figure 5 — Immune related infiltration landscape in testing set. (A)The distribution of immunocytes and immune functions between RIF and control samples in testing set. (B)The abundance differences of different HLA between RIF and control samples in testing set. (C)The abundance differences of different immune checkpoint between RIF and control samples in testing set. * represents P<0.05 compared with the control group,**represents P<0.01 compared with the control group, ***represents P<0.001 compared with the control group. [file Image_5.tif]

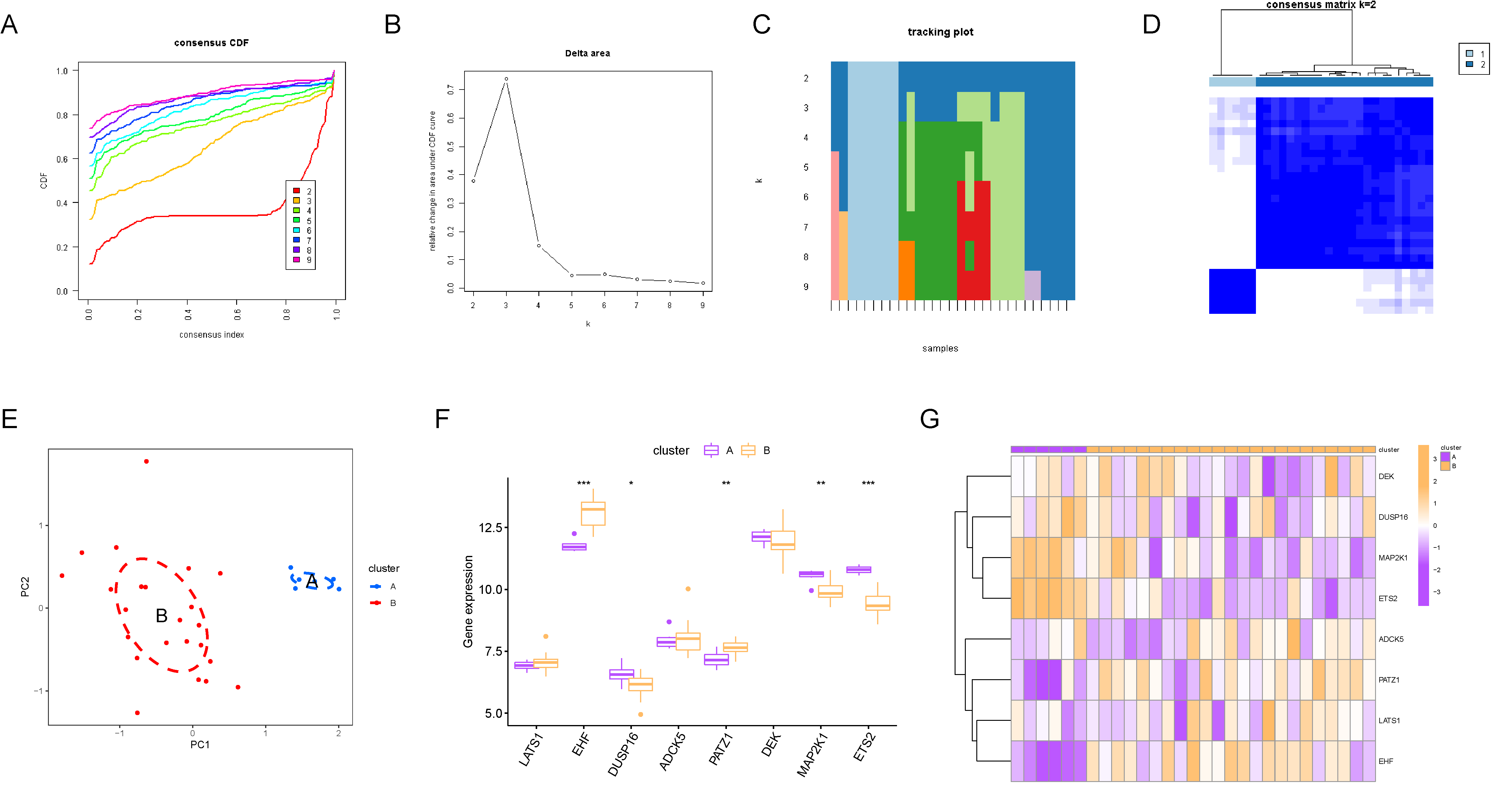

Supplement: Supplementary Figure 6 — Identifying 2 distinct subtypes in RIF by unsupervised clustering based on 8 RIF signature genes. (A) The consensus clustering cumulative distribution function (CDF) for k=2-9. (B) The relative change in area under the CDF curve for k=2-9. (C) Item tracking plot showing the consensus cluster of items (in column) at each k (in row). (D) Consensus matrix plots depicting consensus values on a white to blue color scale ordered by consensus clustering when k= 2. (E) t-SNE plots confirming the classification accuracy of two distinct subtypes across RIF samples. (F–G) Box diagram and heatmap showed the expression level of 8 RIF signature genes in the two subtypes. * represents P<0.05 compared with the cluster A,**represents P<0.01 compared with the cluster A, ***represents P<0.001 compared with the cluster A. [file Image_6.tif]
